# Bones of Contention: Bone Mineral Density Recovery in Celiac Disease—A Systematic Review

**DOI:** 10.3390/nu7053347

**Published:** 2015-05-07

**Authors:** Patricia Grace-Farfaglia

**Affiliations:** 1 Department of Nutritional Sciences, The University of Connecticut, Waterbury, CT 06702, USA; 2 Health and Wellness Promotion, Rocky Mountain University of Health Professions, Provo, UT 84606, USA

**Keywords:** celiac disease, gluten, osteoporosis, diet, physical activity, bone density, nutrient deficiency

## Abstract

Metabolic bone disease is a frequent co-morbidity in newly diagnosed adults with celiac disease (CD), an autoimmune disorder triggered by the ingestion of dietary gluten. This systematic review of studies looked at the efficacy of the gluten-free diet, physical activity, nutrient supplementation, and bisphosphonates for low bone density treatment. Case control and cohort designs were identified from PubMed and other academic databases (from 1996 to 2015) that observed newly diagnosed adults with CD for at least one year after diet treatment using the dual-energy x-ray absorptiometry (DXA) scan. Only 20 out of 207 studies met the inclusion criteria. Methodological quality was assessed using the Strengthening of the Reporting of Observational Studies in Epidemiology (STROBE) statement checklist. Gluten-free diet adherence resulted in partial recovery of bone density by one year in all studies, and full recovery by the fifth year. No treatment differences were observed between the gluten-free diet alone and diet plus bisphosphonates in one study. For malnourished patients, supplementation with vitamin D and calcium resulted in significant improvement. Evidence for the impact of physical activity on bone density was limited. Therapeutic strategies aimed at modifying lifestyle factors throughout the lifespan should be studied.

## 1. Introduction

Celiac disease (CD) is triggered in genetically susceptible individuals by dietary gluten that results in intestinal damage and occurs in 1% of the population in Europe and the United States [1]. Approximately 75% of newly diagnosed patients with celiac disease have low bone mineral density (BMD). And when matched by age and gender to a non-affected population, celiac patients have a 40% greater risk for bone fracture [2]. Even with a silent or extra-intestinal presentation such as dermatitis herpetiformis or dementia, low bone mass is frequently found [3,4,5]. One-third of newly diagnosed cases are over 60 years of age which coincides with a period of an increased risk of falls only worsened by the presence of co-morbidities [6,7]. Women with celiac disease have a much higher rate of fractures during the 10 year period prior to diagnosis and 5 years afterward [8]. The cumulative effects of gluten-induced inflammation, treatment delay, and malabsorption result in lower bone density and bone fragility.

The most effective treatment for celiac disease and related co-morbidities, the gluten-free diet (GFD), is without dispute in the literature. Yet, improvements in BMD after treatment can take as long as two to five years after mucosal recovery [9,10,11,12] Nutritional deficiencies are common during the initial year of treatment for celiac disease, but little data exists on adult micronutrient status and bone mineral density in this population beyond calcium and vitamin D status [13,14,15,16].

The objective of this systematic review is to examine whether a GFD, alone or in combination with other interventions, leads to bone mineral recovery in newly diagnosed adults with celiac disease. A second objective is to identify gaps in the literature that would inform the design of future clinical trials and health intervention studies.

## 2. Methods

### 2.1. Criteria for Considering Studies for this Review

The literature search was limited to English-language articles on untreated and treated adults with celiac disease observed for one year or more. Studies that were exclusively silent or asymptomatic were included if they were part of the sample due to their known bone mineral status. The types of studies considered were cohort, case-control, and randomized controlled designs. Case report and case series were excluded from the analysis. Studies that included co-morbid conditions, such as diabetes or primary hyperparathyroidism, were excluded. Randomized controlled trials and interventions that identified the dose, frequency, type of nutrition supplementation, and physical activity were sought.

### 2.2. Search Strategy for Identification of Studies

A keyword search was accomplished in PubMed (January 1996 to April 2015); Embase; CINAHL plus full text; EBSCO; Scopus; ProQuest Dissertations and Theses, and University of Connecticut library resources (HOMER). The MEDLINE search strategy keywords were “celiac disease” [MeSH]) AND (“bone mineral density” OR “osteoporosis” OR “bone density”) AND (“exercise” OR “physical activity”) AND (“nutrition” OR “gluten-free”) in publication title, abstract, or full-text. The inclusion criteria for this effort were studies with a focus on the treatment of newly diagnosed adults with celiac disease that reported the dependent variable as bone mineral density (BMD) as a dual-energy X-ray absorptiometry (DXA) T-score at baseline and at the annual assessment. Treatment modalities sought included gluten-free diet, physical activity, or vitamin and mineral supplementation. Case studies and papers that did not report DXA data at baseline were excluded.

Key article reference lists were hand searched from the Cochrane database of systematic reviews, meta-analyses, and review articles, as well as papers in (Bone, Gut, Osteoporosis International, and American Journal of Gastroenterology). Finally, journal review articles, and meta-analyses for non-celiac studies of low BMD were reviewed.

### 2.3. Quality Assessments

The clinical trials meeting the criteria are listed in Table 1. Methodological quality was assessed using the STROBE (Strengthening the Reporting of Observational Studies in Epidemiology Statement) recommendations using separate checklists for conference abstracts, case control studies, cohort studies, and cross-sectional studies [17]. The use of a qualitative assessment using a percentage system to categorize the studies as proposed by other authors proved to be unreasonable because of the age of some of the papers, published years before the STROBE recommendations [18]. The final system used was a combination of STROBE (50%–80% fulfilled) and whether missing items left the reader questioning eligibility criteria or bias in the results reported. After 2008, most of the papers fell into the “A” quality category.

### 2.4. Overview of Studies

Three categories of study were identified: GFD; GFD and nutritional supplement; GFD and bisphosphonates; and a combination of GFD, supplement and exercise. The outcome measures in selected articles included: DXA (femoral neck, trochanter and lumbar spine), ligand of receptor activator of NFκB (RANKL) /osteoprotegerin (OPG) ratio, adherence to a GFD, and physical activity level. The clinical trials meeting the criteria are listed in Table 1.

## 3. Results

**Figure 1:**
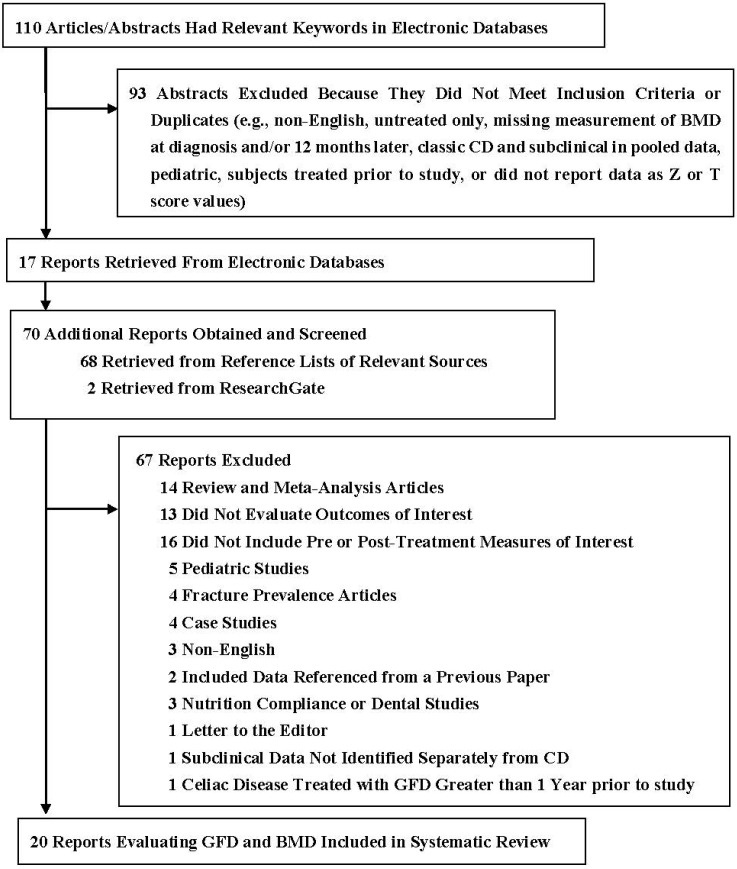
Selection process for studies included in the systematic review.

After the initial screening of articles and abstracts from the initial search strategy, a total of 180 articles were retrieved. The systematic process yielded 20 reports (Figure 1). During the period of this review there was an evolution in diagnosis and classification of CD with greater acceptance of serology test results for screening and monitoring in adults, combined with biopsy for diagnosis [19,20,21]. These developments made study comparisons more difficult because of the clinical heterogeneity between subjects with little or no villous atrophy.

**Table 1:** Studies meeting search criteria.

Author Publication Year	Summary					
	Title	Design	Treatment	Participants	Results	Quality
Corazza (1996) [22]	Reversal of osteopenia with diet in adult coeliac disease	Prospective, case-control	GFD	Gender (M/F) Classical CD 9/10, Mdn age = 26.5 years, Subclinical untreated 11/14, Mdn age = 28.5 years, Control 13/15 (Mdn age = 28)	GFD normalizes BMD in subclinical, but not classical CD.	B
Valdimarsson (1996) [23]	Influence of pattern of clinical presentation and of gluten-free diet on bone mass and metabolism in adult coeliac disease	Prospective, case-control	GFD	*n* = 63, Age range = 17–79 years, M/F = 28/35	After one year taking a GFD bone mineral density increased at all sites (*p* < 0.01). Seven patients with dermatitis herpetiformis had normal BMD, vitamin D & PTH status	B
McFarlane (1996) [24]	Effect of a gluten free diet on osteopenia in adults with newly diagnosed coeliac disease	Prospective, cohort	GFD	*n* = 21 M/F = 14/7, Mean age = 49.7 years (31.0 to 66.1)	Almost half of subjects had osteoporosis. After 1 year of treatment there was significant improvement in BMD	B
Ciacci (1997) [25]	Effects of dietary treatment on bone mineral density in adults with celiac disease: factors predicting response	Prospective, case-control	GFD & calcium	Gender (M/F) 11/30 *n* = 41, M/F = 11/30, Mean age = 34.3	Mean BMD (g cm^−2^) significantly improved by one year after GFD treatment in most, but not all subjects.	B
Mautalen (1997) [26]	Effect of treatment on bone mass, mineral metabolism, and body composition in untreated celiac disease patients.	Prospective, RCT	GFD or GFD plus calcium (1 g day^−1^) & vitamin D (32,000 IU week^−1^)	*n* = 41, M/F = 11/30, Mean age = 34.3 years	Mean BMD (g cm^−2^) significantly improved by one year in most but not all subjects.	B
Kemppainen (1999) [27]	Bone recovery after a gluten-free diet: a 5-year follow-up study	Prospective, cohort	GFD	*n* = 28 newly diagnosed CD patients (9 men, 19 women) recruited from 1990 to 1991. 6 patients withdrew. Women Age = 44.1 ± 13.6 and Men Age = 48.6 ± 12.3 Compliance with the GFD was good: 96% at 1 year and 82% at 5 years.	Bone disease “cured” by 5 years; with most of improvement in the first 12 months.	A
Valdimarsson (1999) [28]	Low circulating insulin-like growth factor 1 in coeliac disease and its relation to bone mineral density	Prospective, case-control, longitudinal	GFD	*n* = 29 CD, Mean age = 41 years, (range 21–66), M/F 8/21, *n* = 29 controls, age and gender matched	BMD and circulating IGF-1 levels are low in adults with untreated CD.	A
Sategna-Guidetti (2000) [29]	The effects of 1-year gluten withdrawal on bone mass, bone metabolism and nutritional status in newly-diagnosed adult coeliac disease patients	Prospective, cohort	GFD	*n* = 86 newly diagnosed CD, M/F = 22/64, mean age M/F = 29/29	GFD leads to significant increase in BMD and IGF-1 levels in postmenopausal women and in patients with incomplete mucosal recovery. Folic acid, albumin and pre-albumin serum levels low for those with incomplete recovery.	A
Taranta (2004) [30]	Imbalance of osteoclastogenesis-regulating factors in patients with celiac disease	Prospective, case-control, longitudinal	GFD	*n* = 25 treated, *n* = 17 untreated, *n* = 17 controls, Treated group mean age = 35.7 ± 7.9 years, Untreated mean age = 43 ± 9.9	Results suggest that bone loss in CD caused by a cytokine imbalance directly affecting osteoclastogenesis. RANKL/osteoprotegerin ratio was increased in patients not on the GFD.	A
Bucci (2008) [31]	PO.7 Physical activity does not influence bone mass density in celiac adult patients	Prospective cohort	Unrestricted and GFD	*n* = 57 adults, age range = 18–45, CD enrolled, 38 completed the study protocol after a 24 months of GFD. High rate of dropout (33%).	GFD induced increase of BMD at femur independently of the amount of reported physical activity, but difference was not significant from baseline to follow-up in the low BMD group. PA was did not differ from baseline at 24 months.	B
Kurppa (2010) [32]	Gastrointestinal symptoms, quality of life and bone mineral density in mild enteropathic coeliac disease: A prospective clinical trial.	Prospective, cohort study	GFD	*n* = 27 (mild enteropathy ), mean age (16–70); *n* = 46 (celiac), mean age 46 (16–70); BMD measured in *n* = 19 (normal villi), *n* = 39 (villus atrophy); *n* = 110 non-celiac controls mean age 49 (24–87)	Osteoporosis or osteopenia was detected in 58% of subjects in the mild enteropathy group and there was a trend towards improved bone mineral density after the treatment.	B
Papamichael (2010) [33]	S2044 effect of a gluten free diet on bone mineral density in patients with celiac disease.	Prospective cohort	GFD/Vitamin D and calcium	*n* = 22 CD patients, M/F = 7/15, mean age = 33 (21–69) years	Women diagnosed with CD because of overt malabsorption had osteoporosis despite supplementation with calcium and vitamin D. At baseline 10 female and all male patients had osteopenia. After 1 year 3 osteoporotic women had osteopenia, while the remaining 19 patients had a normal BMD.	B
Duerksen & Leslie (2011) [34]	Longitudinal evaluation of bone mineral density and body composition in patients with positive celiac serology	Retrospective cohort, database	GFD	Age > 40 years at baseline and testing for CD within 6 mo of baseline DXA test Groups: 37 (seropositive)/214 (controls)	Increase in BMD, BMI, and abdominal fat on GFD. Seropositive *versus* seronegative individuals had greater increases in mean spine BMD (4.6%/year *vs*. 0.7% spine, *p* < 0.0001), hip BMD (3.0%/year *vs*. 0.2% year^−1^ hip, *p* < 0.0001).	A
Vilppula (2011) [35]	Clinical benefit of gluten-free diet in screen-detected older celiac disease patients.	Prospective, cohort study	GFD	*n* = 35, Median age = 61 years (range 52–76), M/F = 15/20	Screen detected older celiac may suffer from subclinical malnutrition, GI symptoms or bone disease. Significant difference between pre & post-treatment femoral and lumbar spine Z scores.	A
Casella (2012) [36]	Celiac disease in elderly adults: clinical, serological, and histological characteristics and the effect of a gluten-free diet	Retrospective, cohorts grouped by age	GFD	M: *n* = 16(A), *n* = 306(B) F: *n* = 43(A), *n* = 860(B) Mean Age at diagnosis, µ ± SD: 70.1 ± 4.3 (65–83 years), 35.2 ± 10.6 (18–64 years)	Prevalence of osteoporosis was 67% in older and 14% in younger male participants and 70% in older and 9% in younger female participants (*p* < 0.001). Lumbar-sacral and femoral T-scores increased significantly during GFD in pooled results of 48 older and younger participants studied before and during GFD.	A
Passananti (2012) [37]	Bone mass in women with celiac disease: role of exercise and gluten-free diet	Prospective, cohort, longitudinal	GFD	48 women of 2-year FU group (Mean age = 35.1 ± 8.7 years) and 47 women of 5-year FU group (Mean age = 35.1 ± 11.3 years)	Improvement in BMD on GFD was significant after 2 years; physical activity was frequently low. No significant relationship was observed between the BMD for 2-year FU and 5-year FU and level of physical activity at diagnosis (*p* > 0.05) for the lumbar spine and for the proximal femur).	B
Szymczak (2012) [38]	Low bone mineral density in adult patients with coeliac disease	Prospective, case-control, longitudinal	GFD plus calcium & alfacalcidol (vitamin D)	*n* = 19 treated, *n* = 16 untreated and 36 controls, second study of supplementation and GFD *n* = 35 CD group M/F = 6/29, Mean age 41.5 ± 13.6 years	Adult CD subjects treated with GFD for one year. They were deficient in calcium, vitamin D, and had lower BMD than controls. Then treated and untreated subjects given diet and supplements for one year. GFD compliant subjects taking supplements had a 35% increase in BMD, but gain was less in non-adherent subjects.	B
Kumar (2013) [39]	Effect of zoledronic acid on bone mineral density in patients of celiac disease: a prospective, randomized, pilot study	Randomized, prospective	GFD group A; and GFD & 4 mg zoledronic acid, calcium (1000 mg) and cholecalciferol (0.6 million units) if serum vitamin D was low, group B	n = 13 (11 completes), and n = 15M/F = 7/6 and 7/8, Mean age 28.2 ± 12.8 years; 25.3 ± 9.1 years	Significant improvement in clinical, biochemical parameters in both groups; GFD with Zoledronic acid was not found to be better than GFD alone after one year.	A
Kurppa (2014) [40]	Benefits of a gluten-free diet for asymptomatic patients with serologic markers of celiac disease.	Prospective, cohort, RCT	Unrestricted and GFD	*n* = 40 randomized to GFD group or unrestricted group, *n* = 20 (M/F = 15/5), *n* = 20 (11/9), Mean age: 42 (21–74) years GFD, Mean age: 42 (21–74) years, unrestricted diet	There were no differences between groups in laboratory test results, BMD (lumbar spine and femur neck), or body composition. Most measured parameters (GI symptoms, psych well-being, and SF-36 QOL) improved when patients in the gluten-containing diet group were placed on GFDs.	A
Pantaleoni (2014) [4]	Bone mineral density at diagnosis of celiac disease and after 1 year of gluten-free diet.	Prospective cohort	GFD	(M/F) *n* = 146/23 Mean age: 38.9 ± 12.6 years, pre/post-menopausal = 104/42	Stratification of patients according to sex and age showed a higher prevalence of low bone mineral density in men older than 30 years and in women of all ages. GFD led to a significant improvement in lumbar spine and femoral neck mean *T*-score value	A

CD: celiac disease; F: female, M: male; BMD: bone mineral density; GFD: gluten-free diet; PA: physical activity; DXA: dual-energy X-ray absorptiometry; BMI: body mass index; FU: follow-up; SF-36 QOL: standard quality of life index; yr.: year; Quality assessment was performed by the author according to STROBE recommendations. RANKL: receptor activator of nuclear factor-κB ligand; PTH: parathyroid hormone; IGF-1: Insulin-like growth factor 1; PA: Physical activity.

Various tests are used to measure bone density, including the DXA, quantitative computed tomography (QTC), photon absorptiometry, and ultrasound. Adult bone mineral loss is categorized into osteopenia and osteoporosis. The World Health Organization (WHO) has established DXA as the best densitometry technique for the measurement of bone density in postmenopausal women, but the sensitivity of the DXA is lower compared to QTC in individuals with celiac disease [41]. A T-score between −1 and −2.5 and ≤2.5 indicates bone mineral loss with a greater than average risk of fracture [12]. Multiple risk factors and the results of the DXA scan are good predictors of relative fracture risk, and prediction is enhanced when co-morbidities, such as celiac disease, are added to the FRAX (WHO Fracture Risk Assessment Tool) index [42].

### 3.1. Diet Therapy

The studies reviewed that reported a dietary compliance measure demonstrated that adherence to the GFD has a positive effect on bone mineral density [4,36,38,43]. The greatest diet treatment gain occurs in the first year of in longitudinal studies that followed individuals for more than 12 months [27,34,37]. But full recovery for adults was mainly achieved after 5 years [27]. Newly diagnosed older adults benefit from the GFD with significant gains in femoral and lumbar spine being reported in the first year of treatment [35,36]. Malabsorption associated malnutrition delayed restoration of BMD in a vitamin D and calcium supplemented high risk group, while less nutritionally compromised individuals in the same study progressed from osteoporosis to osteopenia by one year of treatment [33]. Bone turnover markers Interleukin-18 (IL-18), Interleukin-6 (IL-6), and *N*-terminal telopeptide of procollagen type I were measured in treated (*n* = 25), untreated individuals (*n* = 17), and controls (*n* = 21) [30]. The GFD group had reduced levels of IL-18 and a lower RANKL/osteoprotegerin (OPG) ratio compared to untreated subjects, while the *N*-terminal telopeptide of procollagen type I was comparable to controls. Another study looked at the regulation of osteoclastogenesis and bone turnover in celiac disease in a cohort study of healthy premenopausal female subjects and diet compliant individuals with celiac disease who had no evidence of hypoparathyroidism [44]. The authors reported that the OPG/RANKL ratio was significantly lower in CD patients than in controls (14.8 ± 6.9 *vs.* 19.4 ± 9.2; *p* < 0.05).

Overall, the studies have shown that a GFD for the majority of patients is an effective therapy for long-term bone mineral recovery. The use of intermediate measures such as BMD, quality of life, and bone turnover, is more practical than fracture reduction estimates because these studies followed patients for one to five years. The incidence of fractures in a group of diet compliant participants (*n* = 265, M/F = 422/223) before and 5 years after diagnosis was compared to a cohort of patients with functional gastrointestinal disorders (*n* = 530, M/F = 84/446) [45]. The incidence of fractures declined after treatment (Incidence Rate (IR) = −1.22 events per 1000 patients year^−1^) and Health Risk (HR) of fracture was comparable to controls (HR: 1.28, 95% CI: 0.74–2.21, *p* = ns), thus confirming the long-term benefit of the GFD.

#### 3.1.1. GFD and Nutrient Supplementation

The review identified 3 articles that addressed the treatment effects of vitamin and mineral supplementation on BMD [25,26,33]. The approach of Ciacci, *et al.* was a constant dietary calcium level of 1250 mg daily with diet alone or the addition of a supplement for those who were intolerant to lactose [25]. There was no significant change in vitamin D, urinary calcium increased, and a significant increase in BMD in the lumbar spine, femoral neck, and Ward’s triangle in this study. Non-responders were more likely to be older and menopausal. Mautalen and associates randomized participants to a gluten-free diet condition (*n* = 7) or diet plus calcium (1.0 g day^−1^) and vitamin D (32,000 IU week^−1^) (*n* = 7) [26]. This trial’s results were compromised by non-compliance. Adherent subjects had significant gains in BMD compared to patients that with frequent lapses, and improvement favored the axial compared to the peripheral skeleton. Lastly, Papamichael, *et al.* observed serum 25(OH)D_3_, parathyroid hormone (PTH), and calcium before and after treatment [33]. Only women with severe malabsorption were given supplements, the rest of the study group relied on the GFD and sunshine. In summary, the effect of supplementation depends on the disease severity at diagnosis and environmental exposure to UV radiation. The BMD of the majority of patients improved significantly to adequate vitamin D and calcium in the first year.

Satenga-Guidetti, *et al.* evaluated the pre- and post-treatment nutrient status of subjects (*n* = 86) on a GFD with no supplementation with serum folate, 25OH-D, hemoglobin, transferrin, and albumin measurement, as well as urinary calcium [29]. The researchers observed after a one-year follow-up that 34% of 86 newly diagnosed CD patients had a normal bone mineral density, while 40% had osteopenia and 26% osteoporosis with dietary treatment. There were no differences between gender in bone metabolism markers or most nutritional indices. The only difference was between pre- and post-menopausal women, where BMD and several bone metabolic markers were significantly different. This is in contrast to another study where reduced BMD was found after complete healing of duodenal lesions in one half of their patient sample [46]. The researchers concluded that additional measures beyond dietary compliance and counseling should be taken into account in order to increase BMD.

Treatment failure may actually be the result of a highly processed GFD as it is associated with small bowel bacterial overgrowth (SIBO) [47]. A highly processed GFD has been shown to alter the gut microbiome and increase GI symptoms which leads to an increased permeability of the epithelial barrier in patients with CD [48,49,50]. The health of the gut microbiota and the symptoms related to SIBO impact normalization of bone metabolism. The GFD does not reduce the risk of serious co-morbidities such as small bowel adenocarcinoma, Enteropathy-Associated T-cell Lymphoma (EATL), abortions, myocardial infarction, colonic adenocarcinoma, gastric MALT-lymphoma, ulcerative jejuno-ileitis, Cholangitis, severe non-alcoholicsteato-hepatitis, and autoimmune thrombocytopenia [51].

Nutritional status at time of diagnosis, age, menopausal status, environment, and dietary compliance moderate the effect of dietary supplements on BMD. Controversy exists regarding the amount of supplementation required to reverse malnutrition for key nutrients. A large clinical trial comparing the constant vitamin D and calcium dietary approach to a GFD without supplementation is needed. Because the maintenance of serum vitamin D is improved in the presence of adequate calcium intakes and high levels of physical activity, the study should collect direct measures of total UV radiation exposure and physical activity [52].

#### 3.1.2. GFD and Bisphosphonates

The use of hormone replacement therapy or bisphosphonates, has been shown in healthy pre- and post-menopausal women to be effective in restoring bone mass; and the data suggests that combining these therapies with weight-bearing exercise may have additive effects [53,54]. A small pilot study (*n* = 28) treating CD patients with either GFD and zoledronic acid, or a calcium and cholecalciferol supplemented GFD reported that there were no significant difference in effectiveness [39]. Replication of this study with a larger sample size that includes older adults may be more representative of the adult CD population.

#### 3.1.3. GFD and Physical Activity

Few researchers have studied the effect of physical activity on bone density in this population. Passananti and associates studied two groups of women (20–60 years), and compared baseline measures for the International Physical Activity Questionnaire (IPAQ), fatigue visual analogue scale (VAS), dietary compliance, gastrointestinal symptoms, serum vitamin D, anti-transglutaminase antibodies (U mL^−1^), and BMD in 2 or 5 year follow-up cohorts [37]. Women who were taking oral contraceptives or hormone replacement therapy were excluded. A total of 110 participants were enrolled into the study, but 15 were either lost to follow-up or did not adhere to the GFD. The final sample size of the two cohorts were *n* = 48, *n* = 47, for the 2 and 5 year follow-up cohorts respectively. BMD measurements were taken at the right femur and lumbar spine. Chi-square tests were used for categorical data and ANOVA for continuous measures. Baseline and follow-up (FU) BMD was analyzed by two-sample and pairwise t-tests with a confidence interval set at 95%. Health measures also included BMI, small intestine pathology using Marsh categories for lesion comparisons and age of menarche and menopause. In the 2-year FU group 53.7% of participants reported low physical activity, and 10.6% were highly active (IPAQ = 3). Compared to baseline fatigue in this group, there was no statistical difference. The 5-year FU group was more active, with 46.3% reporting low levels and 14.6% highly active. Yet, the active subjects in in this group were more likely to report fatigue (*p* = 0.039 *post hoc*). The mean IPAQ score for both groups were not significantly different (1.60 ± 0.67 *vs*. 1.87 ± 0.88; *p* > 0.05). There was no significant difference between groups at FU for BMD and no relationship between BMD and intensity of Physical activity (PA) (*p* > 0.05 at the lumbar spine and proximal femur). The researchers concluded that physical activity has a minor role in supporting the bone mineral recovery in celiac patients. It is important to note that the study participants had normal vitamin D levels, but did not meet their dietary needs for calcium.

Di Stefano (2000) observed lifestyle factors in untreated patients with CD and BMD [55]. The participants (*n* = 39) were drawn from a consecutive patient pool with a spectrum of gluten-sensitive disorders: classical celiac, herpetiformis dermatitis, subclinical, or refractory (non-responsive) sprue. The independent variables were gender, smoking status, symptom severity, symptom duration, sunlight exposure, and level of physical activity. PA level was determined by self-report of occupational, recreational (cycling and brisk walking), and activities of daily living on a 0–4 scale of frequency [56]. Predictors of BMD were gender, malnutrition, severity, and PA. Symptom severity predicted low BMD in lumbar and femoral neck (−2.5 ± 0.8, *p* < 0.001). PA was correlated with BMD at the lumbar (*r* = 0.57, *p* < 0.004) and femoral (*r* = −0.71, *p* < 0.004); and bone mineral content (BMC) was correlated with lumbar BMC (*r* = 0.59, *p* < 0.001) and femoral BMC (*r* = 0.58, *p* < 0.001).

Studies concur that fatigue and celiac symptoms impact the frequency and intensity of exercise. The outcome measures, whether biochemical or radiologic, do not take muscle strength or flexibility into account. No direct measure of fitness level or every-day movement has been published. Aside from a study by Gonzalez *et al.* [57], few papers have identified overall body composition changes in spite of observations that treatment results in obesity in some patients [58,59]. In the elderly the impact of exercise on biochemical pathways involved in bone remodeling, such as receptor activator of nuclear factor-κB (RANK) /RANKL/OPG, is positive and particularly responsive to specific types of exercise (e.g., high or low impact exercise and resistance training) [60]. It has been observed that individuals with CD have an altered osteoprotegerin/RANKL ratio, which may also predispose patients to cardiovascular disease [44,61] Future health intervention research in celiac disease should focus on improving wellness, strength, and functional capacity in this population.

A randomized, double-blinded *vs.* placebo parallel study of the effectiveness of *l*-carnitine on fatigue in patients with celiac disease [62]. Participants were given a placebo or 2 g *l*-carnitine daily for 180 days. l-carnitine plays an important role in muscle contraction and energy production. The experimental group experienced an increase in organic cation transporter 2 (OCTN2), a sodium-dependent transporter for carnitine that facilitates carnitine absorption, as well as amines, some vitamins. The l-carnitine treatment group reported significant improvement in fatigue, as evaluated by the VAS scale. The rise in OCTN2 might explain this change. Ultimately, low levels of physical activity in persons with CD may be improved through l-carnitine treatment, which may aid in stimulation of bone mineral deposition.

The specific type of exercise may be a critical factor for improving bone health. Marques and associates studied the effect of resistance and moderate-impact aerobics training protocols on proximal femur BMD, muscle strength, balance, body composition, serum OPG, and RANKL levels in older women (*n* = 71). After eight months of the intervention, only the resistance training group experienced positive changes in BMD and muscle strength [63]. Both protocols had significant effects on functional balance control, a key factor in fall prevention. A later study by this author observed the effect of combined impact training for balance and lower-extremity muscle strength in men and women; the researchers reported improvement in dynamic balance (6.4%), muscle strength (11.0%) and trochanter (0.7%), intertrochanter (0.7%), total hip (0.6%), and lumbar spine BMD (1.7%), while osteocalcin, OPG and RANKL levels remained unchanged [64]. Exercise performed with adequate dose and intensity addresses multiple risk factors for osteoporosis and fractures. Further research on celiac disease lifestyle intervention should begin with known barriers to PA and motivational therapies in other populations [65].

#### 3.1.4. Nutritional Deficiencies, Dietary Sodium, Dysbiosis, and Inflammation

Folate supplementation is an effective treatment for elevated homocysteine in CD [66]. B12 and iron deficiency is a common symptom in untreated CD and in one study it was discovered in 41% of participants [67]. Dahele and Ghosh reported that after oral and parenteral administration of B12, all patients (*n* = 39) had normal serum levels of B12 at one year follow-up. A new role for B12 in bone health has been demonstrated in a mouse model of B12 deficiency demonstrating that deficiency impairs taurine synthesis and enhances Growth hormone-dependent IGF-1 synthesis in the liver; this subsequently increases osteoblast function (Figure 1) [68]. Research in humans should focus on reevaluating vitamin B12 requirements in CD to determine its role in bone remineralization.

Intestinal microbiota has been implicated in the development of CD which may not be corrected after treatment. Due to the low level of phylum *Bacteroides* and abundance of *Firmicutes* in the stools of people with CD, this imbalance has a pro-inflammatory effect by increasing IL-10 cytokine [69]. The GFD did not normalize gut bacteria in one two-year follow-up study of children with CD, and has been shown to induce dysbiosis in healthy adults fed a gluten-free diet for one month [50,70]. Vitamin D metabolite 25(OH)_2_D_3_ has been shown in one vitro study to prime dendritic cells to induce regulatory T (Treg) cells [71]. In that study, the results showed that Dermal dendritic cell-derived IL-10 induce IL-10+ TR1 cells. Gut microbiota also affects T cell differentiation and host susceptibility to autoimmune disease [72]. *Bifidobacterium* genus is low in the stools of celiac patients on a GFD compared to healthy controls consuming a regular diet. This genus has been shown to protect against inflammation and mucosal damage caused by gliadin peptides *in vitro* [73].

A change in diet can bring about long-term improvement in chronic inflammation and malabsorption of nutrients due to alterations in the gut microbiome [74]. Enhanced barrier function due to diet and physical activity was shown in a study of professional elite rugby players eating a diet high in whey protein, fruits, and vegetables during training camp [75]. The DNA from fecal samples showed a rich diversity of organism such as phylum Firmicutes, genus Akkermansia, and fewer Bacteroides than in two less active controls groups comprised of normal weight and obese individuals.

The conclusion of a multivariate analysis of factors predicting recovery in 30 women and 11 men w with CD as that gender (women), pretreatment age, and pretreatment BMD independently predict bone mineralization, especially in the lumbar spine [25]. The regression coefficient for lumbar spine BMD (+0.060 to +0.160 g cm^−2^) did not overlap the 95% confidence interval. The authors identified in their discussion that vitamin D_3_ supplementation may be an important factor that was not addressed in their study. In a double blind placebo controlled study, Fickling and associates (2001) administered 300,000 units of choiecalciferol (vitamin D) by intramuscular injection or saline placebo to newly diagnosed CD patients [76]. Data was collected at 6, 12, and 24 months. Both groups saw improvements in BMD, but no significant difference was found between groups.

A case report of a woman with severe bone mineral loss illustrates the need for individualized treatment of CD [14]. A 37-year-old, sedentary woman diagnosed with CD initially had a low BMD and was prescribed calcium (1000 mg day^−1^) and vitamin D (400 IU day^−1^). After one year a Registered Dietitian-Nutritionist verified diet compliance, serum vitamin D remained low (33 nmol L^−1^), and the dose was subsequently increased to 1000 IU day^−1^. After an 81% increase in the spine and 60% change in the hip at year 3, her bone mass plateaued by year 4. This demonstrates the need to evaluate lifestyle, nutrition, and bone status throughout treatment.

A 10-year study of individuals with CD reported that more than half suffered from vitamin deficiencies in folate, B12 and B6, as evidenced by elevated homocysteine levels, low plasma folate, and B6 [77]. In a Dutch study of newly diagnosed adults (*n* = 80), researchers observed deficiencies in vitamin A, B6, folic acid, B12, and zinc in 67% of participants [78]. B12 and iron deficiency are common in untreated CD and in one study it was discovered in 41% of participants [67]. Dahele and Ghosh reported that after oral and parenteral administration of B12, all patients (*n* = 39) had normal serum levels of B12 at one year follow-up [67]. A new role for B12 in bone health has been shown in a mouse model of B12 deficiency demonstrating that deficiency impairs taurine synthesis and enhances growth hormone-dependent IGF-1 synthesis in the liver; this subsequently increases osteoblast function [68]. Research in humans should focus on reevaluating vitamin B12 requirements in celiac disease to determine its role in bone remineralization.

## 4. Discussion

Our understanding of bone mineral metabolism and the impact of diet and exercise in the treatment of bone mineral loss for people with celiac disease is incomplete. For this review, the author identified published works and most of these were small, non-randomised clinical trials. Because most participants were recruited from University hospitals and specialty treatment centers, the results may be subject to sampling bias. In spite of an exhaustive search only two studies were found using randomised control, and these did not use an intention-to-treat analysis in spite of subject attrition. Studies also lacked uniformity in dietary intake and compliance assessment measures. It is critical to develop a database of individual raw data from studies on bone density and celiac disease so that a meta-analysis of individual participant data can be published [79]. This type of analysis can differentiate treatment effects for different sub-groups, such as non-responders. The incidence of CD continues to rise in all ages groups, creating need for better management of low BMD in adults and elderly [80].

**Figure 2:**
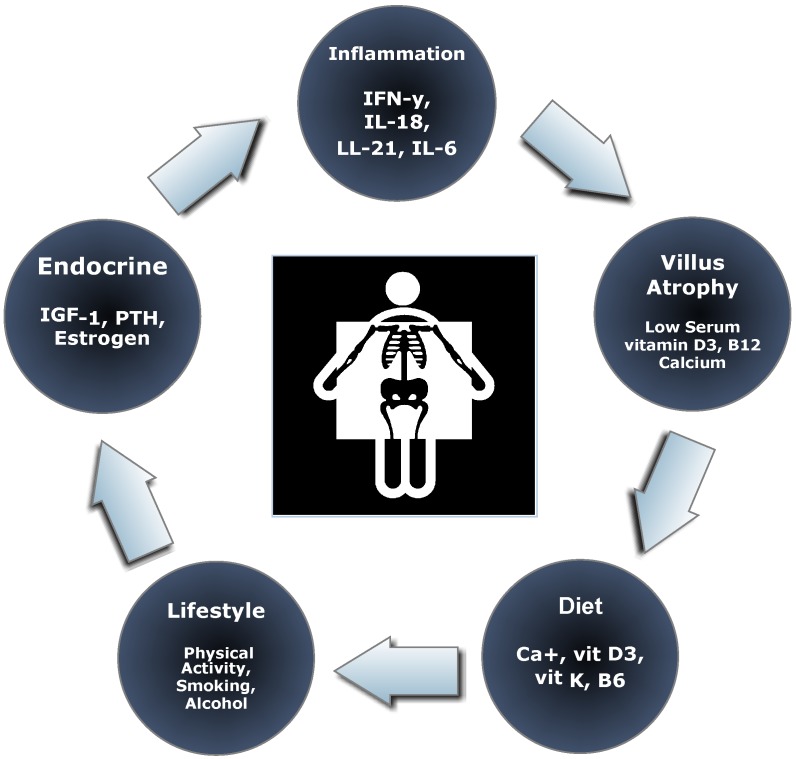
Dysfunctional bone metabolism in celiac disease.

Nutrient malabsorption due to chronic inflammation and villus atrophy are thought to be the major causes of low BMD (Figure 2). In bone, calcium is regulated by parathyroid hormone (PTH), 1,25-dihydroxyvitamin D (Vitamin D3), and calcitonin [81]. Hyperparathyroidism is common in untreated celiac disease and is characterized by high bone turnover and cortical bone loss [82]. In addition, alterations in gallbladder function, exocrine pancreatic insufficiency, and gut permeability reduce the absorption of essential nutrients [83]. BMD is directly related to the extent of villus atrophy which results in malabsorption of calcium, iron, vitamin D, and folic acid [84]. Even after long after the initiation of diet treatment calcium absorption may be reduced [85]. When administered with vitamin D, Vitamin K has been shown to increase BMD in osteoporosis and reduce fracture rates due to its role in calcium balance [86]. 

**Figure 3:**
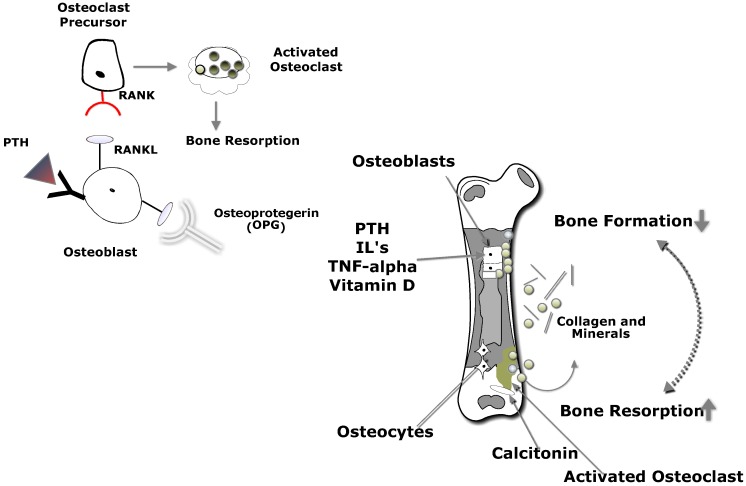
Bone Remodeling. As PTH levels rise, osteoblasts up-regulate the expression of RANKL, which binds to RANK, activating signaling pathways that promote osteoclast differentiation. Osteoblasts secrete osteoprotegerin which protects bone from resorption. Osteoprotegerin binds to RANKL and prevents binding to RANK, therefore the rate stimulation of osteoclastogenesis is reduced. PTH: parathyroid hormone; RANK: receptor activator of nuclear factor-κB; RANKL: receptor activator of nuclear factor-κB ligand.

Bone is dynamic tissue that is continuously going through resorption and absorption of calcium from blood to bone in a process called remodeling (Figure 3). The loss of bone density in CD is caused by an imbalance in osteoclastogenesis and osteoblast activity as reported from a study on newly diagnosed patients compared to individuals on a GFD [30]. The authors reported that proinflammatory factors, *N*-terminal telopeptide of procollagen type I and IL-6, were higher in the untreated CD group, thus suggesting that bone-turnover regulating factors contributed to the reduced bone mass. Other inflammatory factors involved in the pathogenesis of bone metabolism are osteoprotegerin (OPG), a member of the tumor necrosis factor receptor family, and RANKL. RANKL is a cytokine that stimulates osteoclast formation and activation in bone, while OPG acts as a decoy receptor for RANKL, thereby controlling its function. The OPG/RANKL ratio was significantly lower in CD patients in a controlled study of premenopausal women than age matched controls, and the OPG/RANKL ratio was correlated with loss of BMD at the spine [44]. Demographic and lifestyle factors such as age, gender, eating disorders, alcohol abuse, low physical activity, and smoking are also associated with low BMD in CD [55,80,87].

## 5. Conclusions

The most important behavior impacting bone density in celiac disease is diet adherence. The psychological determinants of GFD adherence, such as self-discipline, values, depression, anxiety, and presence of other food intolerances may have an effect on bone rehabilitation [88]. Patients who struggle with personality and psychological barriers to diet compliance may benefit from motivational interviewing techniques early in treatment [89]. Adults diagnosed with celiac disease may have malabsorption of long-standing, therefore may benefit from iron, folate, B12, vitamin D3, vitamin K, calcium, magnesium and docosahexaenoic acid (DHA) therapy [66,77,90]. The nutrient density shortcomings of the gluten-free diet call for the early and individualized services of a registered dietitian-nutritionist with celiac disease expertise in both medical nutrition therapy and lifestyle coaching [91].
